# Development of mobile compatible software for cognitive–communication disorder in individuals with Alzheimer's disease

**DOI:** 10.1111/1460-6984.12941

**Published:** 2023-08-01

**Authors:** Mümüne Merve Parlak, Ayşen Köse, Murat Güç, Özlem Bizpınar Munis

**Affiliations:** ^1^ Department of Speech and Language Therapy, Faculty of Health Sciences Ankara Yıldırım Beyazıt University Ankara Turkey; ^2^ Department of Speech and Language Therapy, Faculty of Health Sciences, Hacettepe University Ankara Turkey; ^3^ Computer Engineering and Software Development, D‐Market Electronic Services and Trade Joint Stock Company (hepsiburada.com) Istanbul Turkey; ^4^ Department of Neurology Ankara Etlik City Hospital Ankara Turkey

**Keywords:** Alzheimer, cognition, cognitive–communication, communication, dementia, software

## Abstract

**Background:**

Alzheimer's disease (AD) is a progressive neurodegenerative disease. Cognitive functions and communication skills worsen as the disease progresses, thereby reducing patients’ independence levels. Therefore, recommending software that can be used at home may be a useful means of slowing down the cognitive and communicative decline in AD.

**Aims:**

To develop software that can be used at home to slow down the cognitive and communicative decline and increase independence in individuals with AD; and to examine the effect of this software on the cognitive communication skills of individuals with AD.

**Methods & Procedures:**

The study was completed in four stages: the development of the application; the evaluation of the participants and their training for the application; their use of the application at home; and the re‐evaluation of the participants. A total of 32 individuals who met the inclusion criteria were included in the study. These individuals were randomly divided into study and control groups, each consisting of a total of 16 participants, including six in the mild stage, six in the moderate stage, and four in the severe stages. The developed software was loaded on tablets and given to the participants in the study group. The participants in the control group only received their pharmacological treatment, while those in the study group received both their pharmacological treatment and used the developed application. All participants were evaluated with the Mini‐Mental State Examination (MMSE) and Language Assessment Test for Aphasia (LATA) before and after application use. A survey was administered to the caregivers of the participants in the study group after the use of the application.

**Outcomes & Results:**

The results revealed a positive change in the cognitive–communication skills of the individuals in the study group, even if they were in the severe stage, according to the LATA and MMSE, as well as the survey. The application led to the greatest improvements in grammar on the LATA and orientation on the MMSE. No rapid cognitive decline was seen in individuals at all stages in the control group.

**Conclusions & Implications:**

In this study, software was developed in Turkish that can be used in every stage of AD as part of a holistic cognitive‐communication intervention programme offering alternative and supportive communication for individuals with AD, even those in the severe stage. Results prove the effectiveness of the developed software on the cognitive–communication skills of individuals with AD.

**What This Paper Adds:**

**What is already known on the subject**

- There are a few (as far as is known, three) applications developed in English for cognitive–communication disorders due to dementia.

**What this study adds to the existing literature**

- In this study, for the first time in Turkish, mobile‐compatible software has been developed for both cognitive and communication disorders that is specific to individuals with AD and can be used by individuals at all stages of the disease. The effects of the application we developed on the cognitive–communication skills of individuals with AD were shown in a randomised controlled trial.

**What are the potential or actual clinical implications of this work?**

- Using the app can help people with all stages of AD keep their cognitive and communication skills and have a better prognosis in terms of cognitive–communication skills, especially grammar and orientation.

## INTRODUCTION

One of the fields of study of speech and language pathologists (SLPs) is cognitive–communication disorders. Cognitive–communication disorder refers to difficulties in any aspect of communication skills as a result of an effect on cognitive functions (American Speech–Language–Hearing Association (ASHA), 2022a; Parlak & Köse, 2023). Cognitive processes include functions such as attention, memory, organization and problem solving or reasoning. Problems with these functions can have an impact on both verbal and non‐verbal communication. Individuals suffering from cognitive–communication disorders may struggle with speaking, listening, reading, writing and pragmatic (social interaction) skills. Furthermore, problems with cognitive–communication can have an impact on an individual's quality of life and daily activities. Dementia, traumatic brain injury, right hemisphere injuries and cerebrovascular events are among the causes of cognitive–communication disorders (ASHA, 2022a, 2022b; Parlak & Köse, 2022). SLPs can use screening scales for cognitive functions, identify areas of impairment through clinical observation and conduct detailed assessments to diagnose and assess such cognitive–communication disorders. As a result of these evaluations, they can determine whether to employ a holistic therapy programme for cognitive–communication or a singular therapy programme for the targeted cognitive subfield (ASHA, 2016; Carrion et al., 2018).

Dementia, one type of cognitive–communication disorder, is a chronic and generally progressive syndrome. Alzheimer's disease (AD) accounts for 60–70% of dementia cases (World Health Organization (WHO), 2017; Parlak et al., 2022a). AD is a progressive neurodegenerative disease in which there is a gradual worsen in cognitive functions, such as orientation, attention, visual/spatial perception and language, with memory being affected initially (Parlak et al., 2022b; Pellicciari & Miniussi, 2018; Parlak et al., 2022c). As the disease progresses, cognitive functions and communication skills worsen, thereby reducing patients’ independence levels. The fact that individuals become increasingly dependent on others also lowers the quality of life of caregivers. The care and support of individuals with AD increase the burden on both families and the healthcare system (Munis et al., 2021; Bayles & Tomoeda, 2007). It is estimated that AD will become a worldwide public health problem by 2050, directly affecting 100 million people (Alzheimer's Association, 2018; Cao et al., 2020). In addition, the Alzheimer's Association stated that according to 2019 data, 16.3 million families and caregivers spent approximately 18.6 million hours on individuals with dementia. This resulted in an average of 21.9 h of care provided by each caregiver per week (2020 Alzheimer's Disease Facts & Figures, 2020). Globally, the cost of the disease is reported to be approximately US$1 trillion per year, and these expenditures will double by 2030 (Cao et al., 2020). However, it is estimated that even preventing a 2‐point decrease in Mini‐Mental State Examination (MMSE) results in individuals with moderate and severe stage AD will save approximately US$3700 per patient per year (O'Hara et al., 2022). Therefore, it is important to establish therapy programmes to support cognitive functions and communication from the early stages of AD (Carrion et al., 2018; Swan et al., 2018). In this way, the level of independence and quality of life of patients can be increased, while care costs and the burden on caregivers can be reduced.

In the literature, there are non‐pharmacologic therapy approaches that can be used in people with AD, and the effects of these therapies on different functions have been shown in studies (Swan et al., 2018; Gibbor et al., 2021; Yu et al., 2022). When therapy effects are considered considering the International Classification of Functioning, Disability and Health (WHO, 2001), some of these different non‐pharmacologic therapy methods are effective in terms of impairment (reality orientation), while others are effective in terms of activity and/or participation. Some therapies are effective in terms of both impairment, activity and participation (cognitive stimulation therapy). According to the American Psychiatric Association (APA), these non‐pharmacological treatments can be cognitive‐, emotion‐, behaviour‐ or stimulation‐based (Carrion et al., 2018). For example, reminiscence therapy is an emotion‐based therapy that has been shown to improve the quality of life and reduce the level of depression in individuals with dementia (Li et al., 2020). Reality orientation therapy is the first non‐pharmacological therapy method based on cognition and is tried to help regain impaired cognitive capacity (Scanland & Emershaw, 1993). The only non‐pharmacological intervention recommended by the National Institute for Health and Care Excellence (NICE) is cognitive stimulation therapy, which is a current cognitive and stimulation‐based method (Bailey et al., 2017). The basic principle of the therapy is to increase and stimulate these functions with the help of certain events and activities targeting certain cognitive functions in individuals (Orrell et al., 2014). Cognitive stimulation therapy has positive results such as strengthening patients’ memory skills and ensuring sustainability, improving coping and adaptation skills, facilitating communication and reducing anxiety and depression (Lök et al., 2020; Woods et al., 2012). In patient groups with progressive cognitive impairment, such as that related to AD, starting therapy immediately after diagnosis is of great importance in terms of reducing the rate of progression and preserving cognitive function (Carrion et al., 2018; Swan et al., 2018). However, many patients are unable to participate in therapies due to transportation issues, low levels of independence and the scarcity of clinicians trained in this field. Therefore, recommending software that can be used at home may be a useful means of slowing down the cognitive and communicative decline in those with this condition. Low‐tech communication books and photo albums are also used to support communication in individuals with AD. Computer‐supported Android applications for cognitive skills such as short‐term memory, executive functions, information processing speed and visual–spatial functions exist as well (Klimova et al., 2018; Armstrong et al., 2010). It has been shown that these applications can improve the quality of life of individuals by increasing their independence (Klimova et al., 2018). However, these applications were mostly developed for healthy elderly individuals, those with mild cognitive impairment and person with cognitive impairment who have mild to moderate AD. The language of use for these applications is English (Akyol & Küçükgüçlü, 2018).

There are also two applications developed in English for dementia‐related cognitive–communication disorders: Computer Interactive Reminiscence and Communication University of Sheffield (CIRCUS) and Computer Interactive Reminiscence and Communication Aid (CIRCA). In these applications, reminiscence therapy for individuals with dementia is combined with the support of communication skills such as initiating and maintaining verbal communication. Unfortunately, these applications have some shortcomings. They do not have a section on the subbranches of cognition, require working with the family and are not designed for the severe stage of dementia (Samuelsson & Ekström, 2019). There is a single application in Malay called My‐MOBAL, which was prepared using reminiscence therapy and cognitive stimulation therapy. However, the effects of this application have been shown in only one patient. While there are some cognitive applications in Turkish, they are designed for people of different ages and are not specific to dementia. In addition, these applications are only for cognitive rehabilitation and do not have a section on communication skills (Akyol & Küçükgüçlü, 2018). The lack of a cognitive and communicative support application that can be used at all stages of the disease in individuals with AD both nationally and internationally constituted the starting point of this study. The plan was to develop an application designed for all stages of the disease using three approaches: emotion‐focused reminiscence therapy; cognition‐focused cognitive stimulation with training for cognition subbranches; and a language section with functional expressions and a communication board (alternative and supportive communication systems). In short, the goals of this study were as follows:

- To develop an application that includes a holistic cognitive–communication intervention programme for both cognitive and communication disorders that can be used in all stages of AD (mild, moderate and severe ).
- To examine the effect of the developed application on patients’ cognitive communication skills.
- To compare the changes among individuals with mild, moderate and severe AD in the study group.

## METHODS

This study was carried out in the Department of Neurology and the Dementia Outpatient Clinic of Health Sciences University Dışkapı Training and Research Hospital. The purpose of the study was explained to the participants and caregivers, and written informed consent forms were obtained using appropriate forms. Ethical approval of the study was obtained by Health Sciences University Dışkapı Training and Research Hospital, with the decision dated 31 May 2021, and numbered 112/01.

### Participants

Inclusion criteria included being diagnosed with AD using DSM‐5 (Diagnostic and Statistical Manual for Mental Disorders, fifth edition) and National Institute on Aging and Alzheimer's Association (NIA‐AA) criteria, being determined to be in the stage of the disease by the clinical dementia rating (CDR) scale, having clinical follow‐up with acetylcholinesterase inhibitors for at least 3 months, being between 60 and 85 years old, and voluntarily participating by themselves or their families.

Exclusion criteria included having a history of additional neurologic disease (stroke, multiple sclerosis, Parkinson's disease, etc.), a history of additional psychiatric disorder (delirium, major depressive disorder, schizophrenia, etc.), having logopenic variant primary progressive aphasia, being followed up in the clinic with acetylcholinesterase inhibitors for less than 3 months, being diagnosed with one of the other types of dementia, and having previously participated in any cognitive and/or communicative therapy.

**FIGURE 1:**
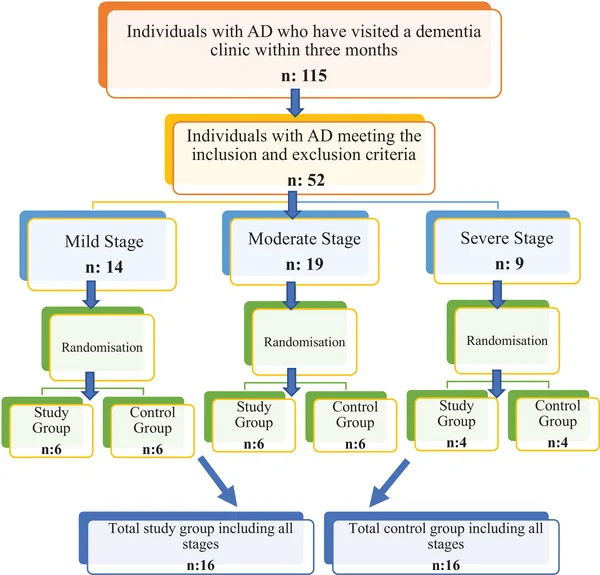
Distribution of individuals in the study. AD, Alzheimer's disease. [Colour figure can be viewed at wileyonlinelibrary.com]

Participants were selected from those who visited the dementia clinic between 21 July and 31 October 2021 and were diagnosed with AD. These individuals’ files were reviewed and participants who met the inclusion and exclusion criteria identified. The required sample size for the study was determined to be the GPower program. When alpha was 0.05, the power value was 0.80 and the effect size was 0.51 (Cohen's *f*), the sample size was determined to be 32 (Gibbor et al., 2021; Faul et al., 2007). Within 3 months, 115 individuals with AD visited the dementia clinic. The files of these individuals were examined, and 52 individuals, consisting of 35 women and 17 men, who met the inclusion and exclusion criteria, were found. According to the post‐test results of these individuals, it was determined that 14 of them had a mild stage, 29 had a moderate stage and nine had an severe stage of AD. Among those who met the inclusion and exclusion criteria, participants for the study and control groups were selected at random. Randomization used the stratification technique. Six mild, six moderate and four severe individuals were selected at random for the control and study groups, for a total of 16 participants. Patients were informed about the study by telephone and invited to the clinic for evaluation. Since there were patients with regular follow‐up, no one refused to participate in the study among the 32 patients. A total of 32 participants took part in this randomised and controlled study. Figure 1 depicts the distribution of the individuals in the study. However, we think that if selection was made from patients who came to the clinic more than 3 months ago, some patients’ caregivers would not be willing to participate in the study. Unfortunately, in our country, only caregivers who are closely interested in the individual with AD bring their relatives to the clinic at frequent intervals. Uninterested caregivers bring them only once a year for the renewal of medication and a nappy report.

### Study design

This randomised controlled trial was carried out in four stages:

Phase 1: Application development.

Phase 2: Participants’ evaluation and practical training.

Phase 3: Participants use the app at home.

Phase 4: Participants’ re‐evaluation.

#### Phase 1: Application development

The entire content and design of the application was made by speech and language therapists. The application's content, subsections and questions to be included in the subsections were all prepared before development. The first author wrote a draft and then the other speech and language therapist reviewed it. Consensus questions and sections were selected. The application's content was created with the goal of focusing on emotion, cognition and communication in people with AD. Cognitive stimulation therapy, reality orientation therapy and reminiscence therapy, which have all been shown to help people with AD, were used as the basis for the content (Gibbor et al., 2021; Woods et al., 2018; Spector et al., 2010; Chiu et al., 2018; Camargo et al., 2015; Khait et al., 2021). Furthermore, because one of the goals of our study was to create a functional application that could be used in all stages of AD, speech and language therapists created a communication board as an alternative and supportive communication system, particularly for individuals in the moderate‐severe stage.

In all sections, an errorless learning strategy was used. According to this strategy, regardless of whether the participant gave the correct or incorrect answer, the questions were designed so that only the correct answer appeared in order for the correct information to be retained and transferred to long‐term memory. Some of the application questions were created by using questions from the book *Therapy Activities in Language and Cognition Disorders* as a model (Çevik et al., 2011). The speech and language therapists created all the other questions. All sections included both written and audio feedback.

The software language of the developed application was Java, and the database was SQLite. The main screen, which is divided into seven sections, was created to be purpose‐oriented and simple to use for cognition subdomains. Orientation, short‐term memory (instant memory), attention and visual spatial functions, executive functions, language, reminiscence and communication board are all application sections (Figure 2.1).

**FIGURE 2:**
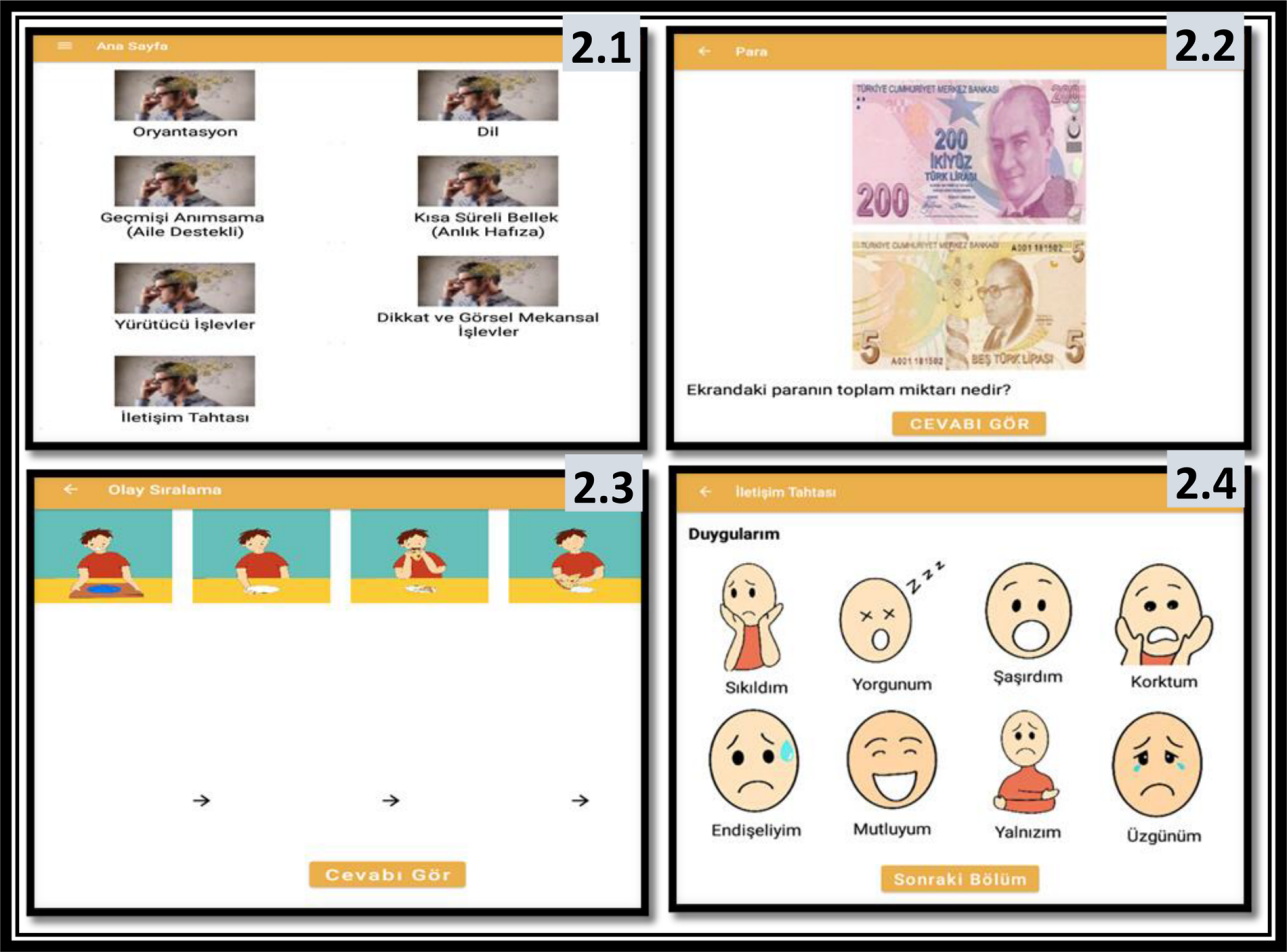
Screenshots of the application. [Colour figure can be viewed at wileyonlinelibrary.com]

##### Orientation section

This section is designed for time and place orientation and is based on reality orientation therapy. When the tab is clicked, the individual is first given a description, then given detailed information, and finally asked the relevant orientation question.

##### Reminiscence section

It is divided into two sections: ‘My Family’ and ‘My Memories’. Based on reminiscence therapy and person orientation, this section aims to maintain and improve long‐term memory skills, particularly in individuals. This section is explained as family supported because the pictures to be uploaded and the information to be entered in the subsections are provided by the families. It is divided into two sections: My Memory pictures and My Memory questions. In the My Memory Pictures section, individuals are asked to upload pictures that will remind them of their past memories, such as weddings, births, military service, etc., and to fill in the description of the picture. In the My Memory Questions section, there are questions such as date of birth, place of birth, names of mother, father, children, grandchildren, favourite friends and cities visited before.

##### Executive functions section

This section aims to improve a person's functionality and independence in daily activities and includes some activities used in cognitive stimulation therapy. There is money (Figure 2.2), event sequencing and problem‐solving subsections (Figure 2.3).

##### Language section

There are three subsections: functional expressions (e.g., what do you say when someone says, ‘See you soon?’), naming (what do you see in the picture?) and showing what is said. This section is specifically designed to develop communicative independence. In the showing what is said subsection, there are questions about its name is showing what is said (e.g., show the spoon), its function is showing what is said (e.g., which one is used for writing?), categorization (e.g., which one is a fruit?) and object–verb relations (e.g., which one is an event related to a nail?).

##### Short‐term memory section

This section consists of two subsections: auditory memory and visual memory. Auditory memory subsection: There are questions about mixed names of objects, animals, etc.; numbers; human names; all mixed. Visual memory subsection: There are questions about number, shape, a human picture and colour.

##### Attention and visual spatial functions section

There are two subsections to the questions: finding what is the same and finding what is different. Numbers, shapes and colours are among the questions.

##### Communication board section

This section is designed especially for advanced individuals to show their basic needs, pain and emotional states. The main headings are: my emotions, my health status, my wishes, my food and drinks, and my belongings. On each page there are eight photographs with descriptions, and when clicked on, they give feedback in an audible way as if spoken by the individual. ‘I'm hungry’, ‘I need water’, etc. In this section, there are a total of 88 pictures in the form of drawings or photographs. Figure 2.4 shows the emotions section in the communication board of the application.

#### Pilot implementation

A pilot study was conducted with eight healthy individuals (four women and four men) over 65 years of age (mean age = 74.63 ± 3.34 years) and the deficiencies in the application were identified. In line with the feedback, the pictures and text were enlarged. Three pictures from the naming section and two pictures from the health status section were removed because they were difficult to understand.

There are a total of 840 questions or pictures in the application, excluding the memory pictures subsection of the reminiscence section. The number of questions increases according to the number of pictures added by the families in the memory pictures section.

#### Phase 2: Participants evaluation and implementation training

In the neurology clinic, participants from both the study and control groups were evaluated. The neurologist administered the Standardized Mini‐Mental State Examination (MMSE) for cognitive assessment, and an SLP who did not know the patient's stage or the MMSE result administered the Language Assessment Test for Aphasia (LATA) for language and communication assessment. Since there is no valid and reliable dementia‐specific communication scale in Turkish, the use of LATA was chosen.

With MMSE, five subfields of cognitive functions were evaluated: orientation, registration (early recall), attention and calculation, recall (late recall), and language. Maviş and Toram created the Turkish standardised language assessment test LATA in 2012 (Toğram & Maviş, 2012). LATA is frequently used not only in people with aphasia but also in people with other acquired language disorders (Yaşar et al., 2016). With LATA, eight subfields were evaluated, including speech fluency, auditory comprehension, repetition, naming, reading, word actions, grammar and writing.

##### Study group

After the initial evaluation, the participants and their caregivers were shown how to open the application, its sections and how to use it. Each section was tested three times with both the patient and the family (Figure 3.1). An SMS message was sent to the families about how to use the application and the duration of the application.

**FIGURE 3:**
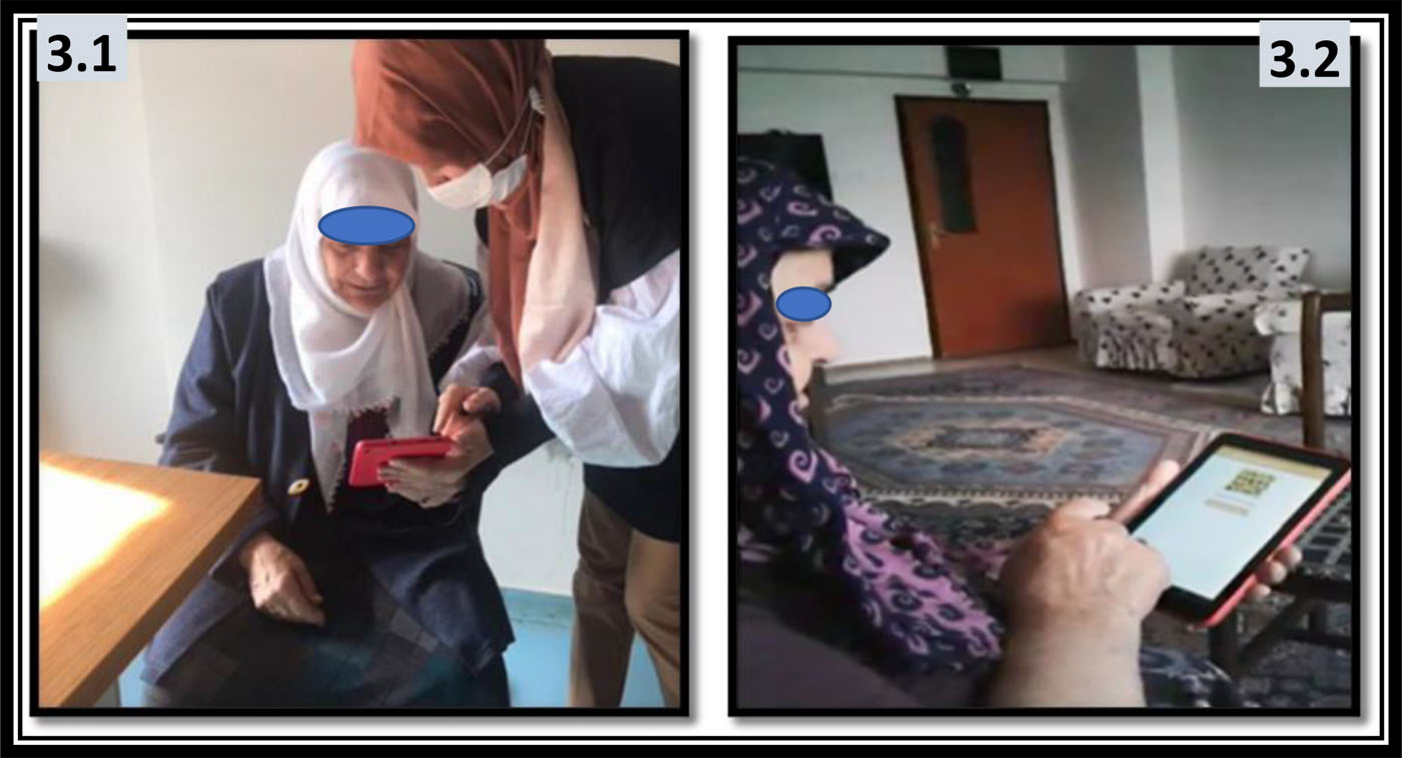
Demonstration of application use, and use of the application at home. [Colour figure can be viewed at wileyonlinelibrary.com]

##### Control group

After the first evaluation, the participants were told they would be re‐evaluated in 7 weeks and could get therapy with the app after the last evaluation if they wanted.

#### Phase 3: Participants using the app at home

##### Study group

The researchers purchased 16 tablets of the Everest Everpad SC‐725 brand, Android 8.1, IPS screen and 7‐inch. Participants were given a tablet with an app pre‐installed. All other applications (internet, games, other applications, etc.) were removed, leaving only the application developed on this tablet accessible. The tablet is configured to display only the app icon on the home screen. On all tablets, the volume was set to maximum, the screen touch volume was enabled and the screen light off time was set to 1 h. Individuals in the study group used the developed application in addition to their ongoing pharmacological treatment for 7 weeks. Figure 3.2 shows a mid‐stage participant using the app at home.

Studies of therapy and training for people with dementia have delivered interventions ranging from 2 weeks (Gagnon & Belleville, 2012) to 2 years (Amieva et al., 2016). Because studies of these various durations have yielded similar positive results, it has been stated that the duration makes no difference in terms of these non‐pharmacologic treatments (Carrion et al., 2018; Swan et al., 2018). Positive changes in multiple areas such as cognition, depression and quality of life in individuals with AD have been observed in therapies for more than one subsection of cognition if they are studied for more than 6 weeks (Swan et al., 2018; Carrion et al., 2018). The duration of use of the application was determined in our study to be 7 weeks because the cognitive stimulation therapy on which the application is based is used for 7 weeks in the literature (Lök et al., 2020). Furthermore, the average duration of reminiscence therapy, on which the application is based, is 6 weeks (Lök et al., 2019). Because cognitive subfields, in addition to reminiscence therapy, were studied, the duration of use of our application was determined to be 7 weeks.

Individuals with AD and their caregivers were asked to practice the reminiscence section 2 days a week for 30 min because reminiscence therapy is a therapy method that is used 2 days a week for 30–60 min. Furthermore, while the maximum duration of therapies administered in the clinic is 5–6 days per week for 1–2 h, applications are administered 5–7 days per week for 15–30 min. Therefore, the participants were asked to spend 1 h each day, 3 days a week, working on the application's subsections. As a result, the participants received 5 days of therapy with the application and the reminiscence section. Furthermore, participants in the severe stage were asked to use the communication board to express their needs, pain and emotional states as needed. The participants’ caregivers were contacted on a weekly basis to obtain information about their use of the application and the number of hours they worked per day. It was attempted in this manner to ensure the application's continued use.

##### Control group

The control group only received pharmacological treatment. Those who volunteered after this study received cognitive–communication therapy with the application.

#### Phase 4: Re‐evaluation of participants

**FIGURE 4:**
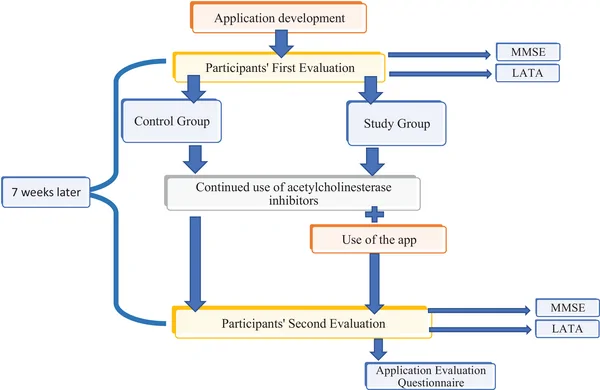
Study design. LATA, Language Assessment Test for Aphasia; MMSE, Mini‐Mental State Examination. [Colour figure can be viewed at wileyonlinelibrary.com]

Seven weeks later, all study and control group participants were re‐evaluated using the MMSE and LATA tests used in the initial evaluation. The Cognitive Communication Disorder Application Evaluation Questionnaire, consisting of 13 questions, was administered to caregivers or family members of study group participants during the final evaluation (Table 4). The purpose of this questionnaire was to assess the application's ease of use, understandability, design and satisfaction factors. The questionnaire employed a 5‐point Likert scale (5 = strongly agree, 4 = agree, 3 = undecided, 2 = disagree, 1 = strongly disagree). There is also an open‐ended comment section, such as ‘If you have any comments you would like to add, please indicate.’ where participants can share their thoughts on the application and its results. A summary of the design of the study is shown in Figure 4.

### Data analysis

Frequency (number–percentage) values of the study participants’ demographic information were calculated. The Shapiro–Wilks test was used to ensure that all continuous variables followed a normal distribution. The chi‐square test and one‐way analysis of variance (ANOVA) were used to compare demographic variables based on stages according to groups.

The independent sample *t*‐test and chi‐square test were used to compare variables specified on the basis of groups (study control). The independent sample *t*‐test was used to compare age and years of education in the study and control groups based on the stages of AD (e.g., mild stage in the study group and mild stage in the control group). The dependent sample 2 test was used to compare the before and after values of each group (e.g., study group mild stage first evaluation and study group mild stage last evaluation). One‐way ANOVA was used to determine whether the total score and subdimension scores of the questionnaire administered to caregivers showed statistically significant differences based on AD stages. The questionnaire scores were given descriptive statistics.

IBM SPSS Statistics for Windows, Version 21.0, was used. The statistical significance level was accepted as *p* < 0.05.

## RESULTS

### Sociodemographic characteristics of the participants

Of the individuals in the study, 40.6% (*n* = 13) were male and 59.4% (*n* = 19) were female. 37.5% were in the mild stage, 37.5% were in the moderate stage, and 25% were in the severe stage. The mean age of the participants in the study group was 75.00 ± 6.38 years and 74.63 ± 6.60 years in the control group (Table 1).

**TABLE 1:** Sociodemographic characteristics.

			Groups		Test statistics	
Variables			Study group	Control group	*t*, *χ* ^2^	*p*
			*n* (%)	*n* (%)		
**Age**	Total participants, including all stages (74.81 ± 6.39)		75.00 ± 6.38	74.63 ± 6.60	0.163	0.871
**Mean ± SD**	Mild stage		74.67 ± 6.40	73.17 ± 8.90	0.335	0.745
	Moderate stage		74.33 ± 6.83	75.33 ± 5.68	0.276	0.788
	Severe stage		76.50 ± 7.32	75.75 ± 4.99	0.169	0.871
**Number of people**	Total participants		16 (50.0)	16 (50.0)	–	–
	Mild stage		6 (50.0)	6 (50.0)		
	Moderate stage		6 (50.0)	6 (50.0)		
	Severe stage		4 (50.0)	4 (50.0)		
**Years of education**	Total participants		3.19 ± 2.90	3.19 ± 2.31	0.000	1.000
	Mild stage		4.00 ± 2.96	4.17 ± 2.04	0.113	0.912
	Moderate stage		2.17 ± 2.48	2.17 ± 2.48	0.000	1.000
	Severe stage		3.50 ± 3.69	3.25 ± 2.36	0.114	0.913
**Gender**	Total participants	Male	7 (53.8)	6 (46.2)	0.129	0.719
		Female	9 (47.4)	10 (52.6)		
	Mild stage	Male	3 (60.0)	2 (40.0)	0.343	0.500
		Female	3 (42.9)	4 (57.1)		
	Moderate stage	Male	2 (50.0)	2 (50.0)	0	0.727
		Female	4 (50.0)	4 (50.0)		
	Severe stage	Male	2 (50.0)	2 (50.0)	0	0.727
		Female	2 (50.0)	2 (50.0)		

There were no statistically significant differences in age, gender and years of education between the study and control groups in different stages of AD and in all participants (mild, moderate, advanced and all participants) (Table 1).

### Participants’ MMSE outcomes

**FIGURE 5:**
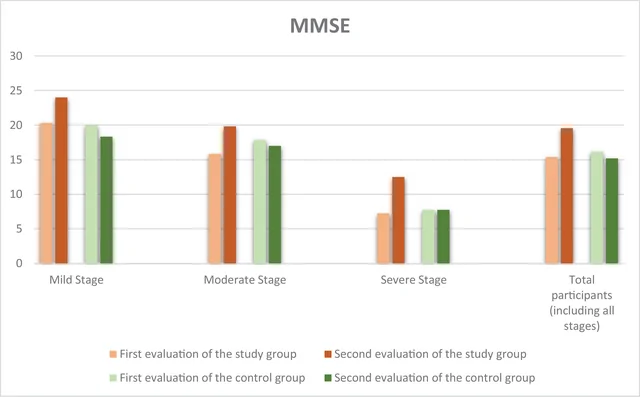
Change in participants’ total Mini‐Mental State Examination (MMSE) results. [Colour figure can be viewed at wileyonlinelibrary.com]

After 7 weeks, the MMSE total score increased from 15.38 ± 5.80 to 19.56 ± 5.76 in the study group and decreased from 16.13 ± 5.65 to 15.19 ± 5.46 in the control group (Table 2 and Figure 5).

**TABLE 2:** Participants’ MMSE outcomes.

	Mild stage			Moderate stage			Severe stage			Total participants		
MMSE	Study group, mean ± SD	Control, mean ± SD	Test statistics, *t*; *p*	Study group, mean ± SD	Control, mean ± SD	Test statistics, *t*; *p*	Study group, mean ± SD	Control, mean ± SD	Test statistics, *t*; *p*	Study group, mean ± SD	Control, mean ± SD	Test statistics, *t*; *p*
***Orientation***												
**First evaluation**	6.50 ± 1.87	6.17 ± 1.72	0.321; 0.755	5.33 ± 1.03	4.67 ± 1.21	1.026; 0.329	3.00 ±1.41	2.00 ± 1.41	1.000; 0.356	5.19 ± 1.97	4.56 ± 2.15	0.855;
**Second evaluation**	8.67 ± 1.21	5.83 ± 1.72	3.296; 0.008**	6.83 ± 1.72	4.17 ± 1.32	3.002; 0.013*	4.00 ± 2.44	2.00 ± 1.63	1.359; 0.223	6.81 ± 2.48	4.25 ± 2.11	3.144; 0.004**
**Test statistics *t*; *p***	3.606; 0.015*	1.000; 0.363		2.666; 0.045*	1.075; 0.076		1.732; 0.182	0;1		4.779; < 0.001***	1.775; 0.096	
***Registration***												
**First evaluation**	3.00 ± 0	3.00 ± 0	–	2.67 ± 0.51	2.67 ± 0.51	0;1	1.50 ± 1.29	1.00 ± 1.41	0.522; 0.620	2.50 ± 0.89	2.38 ± 1.08	0.355; 0.725
**Second evaluation**	3.00 ±0	2.83 ±0.40	1.000; 0.341	3.00	2.83 ± 0.40	1.000;0.341	2.25 ± 0.95	2.25 ± 0.95	0;1	2.81 ± 0.54	2.69 ± 0.60	0.616; 0.542
**Test statistics *t*; *p***	–	1.000; 0.363		1.581; 0.175	1.000; 0.363		1.000; 0.391	1.987; 0.141		1.576; 0.136	1.431; 0.173	
***Attention and calculation***												
**First evaluation**	3.17 ± 1.32	3.17 ± 1.94	0;1	1.67 ± 2.06	4.00 ± 0.89	2.539; 0.029	0	0.25 ± 0.50	1.000; 0.356	1.81 ± 1.90	2.75 ± 1.98	1.364; 0.183
**Second evaluation**	4.17 ± 1.60	2.33 ± 2.06	1.718; 0.117	2.50 ± 2.42	3.67 ± 0.81	1.115; 0.291	0	0	–	2.50 ± 2.36	2.25 ± 1.94	0.326; 0.747
**Test statistics *t*; *p***	2.739; 0.041*	1.623; 0.042*		2.076; 0.093	1.000; 0.363		–	1; 0.391		3.149; 0.007**	2.739; 0.015*	
***Recall***												
**First evaluation**	0.33 ± 0.51	0.50 ± 0.83	0.415; 0.687	0.14 ± 0.40	0.17 ± 0.40	0; 1	0	0	–	0.20 ± 0.41	0.25 ± 0.57	0.355; 0.725
**Second evaluation**	0.33 ± 0.51	0.33 ± 0.81	0;1	0.14 ± 0.40	0	1; 0.341	0	0	–	0.20 ± 0.41	0.13 ± 0.50	0.453; 0.654
**Test statistics *t*; *p***	0; 1	1.000; 0.363		–	1.000; 0.363		–	–		0.000; 1.000	1.464; 0.164	
***Language***												
**First evaluation**	7.33 ± 0.81	7.17 ± 0.75	0.368; 0.721	6.00 ± 0.63	6.33 ± 0.81	0.791; 0.448	2.75 ± 1.25	4.50 ± 1.29	1.941; 0.100	5.69 ± 2.024	6.19 ± 1.37	0.817; 0.420
**Second evaluation**	7.83 ± 0.98	6.83 ± 0.75	1.978; 0.076	7.33 ± 0.81	6.33 ± 1.21	1.677; 0.129	6.25 ± 0.95	4.25 ± 1.50	2.248; 0.066	7.25 ± 1.06	6.00 ± 1.50	2.712; 0.011*
**Test statistics *t*; *p***	1.168; 0.296	0.791; 0.465		4.000; 0.010*	0;1		2.581; 0.001**	0.522; 0.638		4.283; 0.001**	0.899; 0.383	
***Total score***												
**First evaluation**	20.33 ± 1.75	20.00 ± 3.79	0.195; 0.849	15.83 ± 2.78	17.83 ± 0.98	1.658; 0.128	7.25 ± 3.59	7.75 ± 2.21	0.237; 0.821	15.38 ± 5.80	16.13 ± 5.65	0.370; 0.714
**Second evaluation**	24.00 ± 2.60	18.33 ± 4.96	2.474; 0.033*	19.83 ± 4.49	17.00 ± 1.67	1.448; 0.195	12.50 ± 3.87	7.75 ± 1.89	2.204; 0.070	19.56 ± 5.76	15.19 ± 5.46	2.203; 0.035*
**Test statistics *t*; *p***	2.583; < 0.001***	1.814; 0.129		4.899; 0.004**	1.387; 0.224		10.967; 0.002**	0; 1		11.055; < 0.001***	2.076; 0.055	

The mild stage study group's participants’ orientation (*p* = 0.015), attention‐calculating (*p* = 0.041) and total MMSE (*p* = 0.001) scores all showed statistically significant increases, whereas the attention‐calculating score of the control group's participants showed a statistically significant decline (*p* = 0.042). Participants in the moderate stage study group experienced statistically significant increases in their orientation (*p* = 0.045), language (*p* = 0.010) and total MMSE (*p* = 0.004) scores. In the severe stage study group, the participants’ language (*p* = 0.001) and total MMSE (*p* = 0.002) scores improved statistically significantly (Table 2).

For all participants, the orientation (*p* = 0.001), attention‐calculating (*p* = 0.007), language (*p* = 0.001) and total MMSE (*p* = 0.001) scores increased statistically significantly in the study group, whereas the attention‐calculating (*p* = 0.015) score decreased statistically significantly in the study group and a statistically significant difference was found between the study and control groups in terms of orientation (*p* = 0.004), language (*p* = 0.011) and total MMSE (*p* = 0.035) scores (Table 2).

### Participants’ LATA outcomes

**FIGURE 6:**
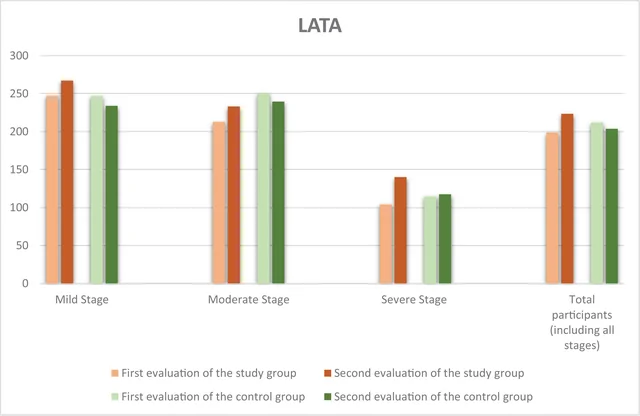
Change in participants’ total Language Assessment Test for Aphasia (LATA) results. [Colour figure can be viewed at wileyonlinelibrary.com]

All LATA subsection scores except writing and the LATA total score increased from 198.63 ± 76.11 to 223.18 ± 70.43 statistically significantly for all participants in the study group, including all stages (*p* < 0.05) (Table 3 and Figure 6).

**TABLE 3:** Participants’ LATA outcomes.

	Mild stage			Moderate stage			Severe stage			Total participants		
LATA	Study group, mean ± SD	Control group, mean ± SD	Test statistics *t*; *p*	Study group, mean ± SD	Control group, mean ± SD	Test statistics *t*; *p*	Study group, mean ± SD	Control group, mean ± SD	Test statistics *t*; *p*	Study group, mean ± SD	Control group, mean ± SD	Test statistics *t*; *p*
***Speech fluency***												
**First evaluation**	28.17 ± 2.92	27.33 ± 4.13	0.403; 0.695	24.17 ± 2.22	27.50 ± 4.32	1.678; 0.124	16.25 ± 2.36	15.25 ± 8.05	0.238; 0.820	23.69 ± 5.33	24.38 ± 7.38	0.302; 0.765
**Second evaluation**	30.00 ± 1.67	25.83 ± 4.44	2.148; 0.057	26.50 ± 2.25	26.33 ± 3.44	0.099; 0.923	13.00 ± 7.78	13.00 ± 7.78	1.565; 0.169	26.06 ± 4.69	22.81 ± 7.54	1.462; 0.154
**Test statistics *t*; *p***	2.607; 0.048*	1.964; 0.107		4.183; 0.009**	1.941; 0.110		2.472; 0.090	3.000; 0.058		5.216; < 0.001***	3.930; 0.001**	
***Auditory comprehension***												
**First evaluation**	57.67 ± 5.98	53.33 ± 7.65	1.092; 0.301	45.67 ± 7.00	47.33 ± 9.83	0.338; 0.742	20.25 ± 13.79	28.50 ± 12.28	0.893; 0.406	43.81 ± 17.10	44.88 ± 13.58	0.195; 0.847
**Second evaluation**	63.00 ± 4.69	52.17 ± 8.03	2.852 0.017*	54.33 ± 8.11	45.50 ± 9.39	1.743; 0.112	35.25 ± 9.53	30.75 ± 9.53	0.434; 0.679	52.81 ± 14.88	44.31 ± 11.97	1.779; 0.085
**Test statistics *t*; *p***	2.902; 0.034*	1.941; 0.110		3.281; 0.022*	1.701; 0.150		2.554; 0.084	0.279; 0.798		4.524; < 0.001***	0.296; 0.771	
***Repetition***												
**First evaluation**	16.50 ± 2.95	17.00 ± 2.89	0.296; 0.773	13.00 ± 2.36	15.83 ± 3.43	1.665; 0.127	9.25 ± 6.80	10.00 ± 6.78	0.156; 0.881	13.38 ± 4.74	14.81 ± 4.94	0.839; 0.408
**Second evaluation**	18.50 ± 1.22	15.17 ± 3.25	2.351; 0.041*	16.33 ± 2.42	14.00 ± 2.09	1.784; 0.105	11.75 ± 6.07	10.50 ± 1.73	0.396; 0.706	16.00 ± 4.14	13.56 ± 3.03	1.898; 0.067
**Test statistics *t*; *p***	1.464; 0.203	4.568; 0.006**		3.071; 0.028*	3.051; 0.028*		2.402; 0.096	0.197; 0.856		3.882; 0.001**	1.855; 0.083	
***Naming***												
**First evaluation**	40.50 ± 4.03	36.17 ± 8.13	1.169; 0.270	38.50 ± 2.34	37.50 ± 2.95	0.650; 0.530	19.25 ± 14.99	22.50 ± 5.44	0.407; 0.698	34.44 ± 11.62	33.25 ± 8.50	0.330; 0.744
**Second evaluation**	42.83 ± 1.83	34.67 ± 8.35	2.338; 0.042*	40.17 ± 2.13	36.83 ± 2.31	2.591; 0.027*	25.00 ± 12.93	23.25 ± 4.92	0.253; 0.809	37.38 ± 9.59	32.63 ± 7.88	1.531; 0.136
**Test statistics *t*; *p***	1.988; 0.104	2.087; 0.091		1.536; 0.185	0.877; 0.421		1.910; 0.152	0.168; 0.877		3.008; 0.009**	0.575; 0.574	
***Reading***												
**First evaluation**	38.40 ± 8;.41	39.40 ± 2.07	0.258; 0.803	33.00 ± 11.35	43.33 ± 1.52	1.562; 0.193	18.00 ± 16.09	11.67 ± 9.81	0.582; 0.592	31.36 ± 13.60	32.91 ± 14.50	0.258; 0.799
**Second evaluation**	42.40 ± 5.94	37.40 ± 4.21	1.534; 0.164	34.33 ± 12.50	41.33 ± 3.05	0.942; 0.400	22.33 ± 17.61	9.33 ± 11.01	1.084; 0.339	34.73 ± 13.52	30.82 ± 15.05	0.641; 0.529
**Test statistics *t*; *p***	2.760; 0.051	1.907; 0.129		2.000; 0.184	1.309; 0.321		1.522; 0.268	2.646; 0.118		3.355; 0.007**	3.429; 0.006**	
***Grammar***												
**First evaluation**	14.67 ± 3.67	12.67 ± 6.40	0.663; 0.522	13.67 ± 2.73	12.33 ± 3.20	0.776; 0.456	6.50 ± 4.72	8.75 ± 6.29	0.572; 0.588	12.25 ± 4.83	11.56 ± 5.27	0.384; 0.704
**Second evaluation**	17.67 ± 3.20	10.00 ± 4.85	3.227; 0.009**	16.67 ± 2.94	11.33 ± 3.38	2.911; 0.016*	8.75 ± 4.99	9.25 ± 4.03	0.156; 0.881	15.06 ± 5.06	10.31 ± 3.96	2.954; 0.006**
**Test statistics *t*; *p***	3.105; 0.027*	2.329; 0.067		4.743; 0.005**	2.236; 0.076		9.000; 0.003**	0.258; 0.813		6.688; < 0.001***	1.806; 0.091	
***Word actions***												
**First evaluation**	19.17 ± 0.983	17.67 ± 2.58	1.330; 0.213	16.17 ± 1.60	18.17 ± 1.94	1.947; 0.080	9.00 ± 6.16	10.25 ± 4.50	0.328; 0.754	15.50 ± 5.06	16.00 ± 4.39	0.298, 0.767
**Second evaluation**	20.00 ± 0	15.83 ± 1.16	8.730; < 0.001***	19.50 ± 1.22	17.83 ± 2.04	1.715; 0.117	11.50 ± 5.19	10.00 ± 4.32	0.444; 0.673	17.69 ± 4.42	15.13 ± 3.96	1.726; 0.095
**Test statistics *t*; *p***	2.076; 0.093	3.051; 0.028*		5.000; 0.004**	0.598; 0.576		2.887; 0.063	0.174; 0.873		4.973; < 0.001***	1.849; 0.084	
***Writing***												
**First evaluation**	30.60 ± 17.15	32.60 ± 7.02	0.241; 0.815	25.67 ± 13.57	30.33 ± 15.88	0.387; 0.719	6.67 ± 6.11	7.00 ± 6.24	0.066; 0.950	22.73 ± 16.52	25.00 ± 14.58	0.342; 0.736
**Second evaluation**	32.20 ± 15.78	32.40 ± 7.63	0.026; 0.980	26.67 ± 13.01	30.33 ± 15.04	0.319; 0.765	14.00 ± 12.53	6.33 ± 5.68	0.965; 0.389	25.73 ± 15.08	24.73 ± 14.67	0.158; 0.876
**Test statistics *t*; *p***	2.359; 0.078	0.408; 0.704		1.732; 0.225	0; 1		1.367; 0.305	2.000; 0.184		1.944; 0.081	1.000; 0.341	
***Total score***												
**First evaluation**	247.00 ± 40.32	246.80 ± 18.29	0.010; 0.992	212.66 ± 24.13	250.33 ± 22.81	1.965; 0.121	104.00 ± 74.53	114.66 ± 55.33	0.199; 0.852	198.63 ± 76.11	211.72 ± 68.83	0.423; 0.677
**Second evaluation**	267.00 ± 28.15	233.80 ± 20.90	2.117; 0.067	233.00 ± 35.59	239.33 ± 25.89	0.249; 0.815	140.33 ± 81.94	117.33 ± 16.25	0.477; 0.658	223.18 ± 70.43	203.54 ± 58.59	0.711; 0.485
**Test statistics *t*; *p***	3.101; 0.036*	4.568; 0.010*		2.853; 0.104	3.667; 0.067		2.603; 0.121	0.112; 0.921		4.798; 0.001**	1.340; 0.210	

The mild stage study group's participants’ speech fluency (*p* = 0.048), auditory comprehension (*p* = 0.034), grammar (*p* = 0.027) and total LATA (*p* = 0.036) scores increased statistically significantly, whereas repetition (*p* = 0.006) and word action (*p* = 0.028) scores decreased statistically significantly in the control group's participants. Speech fluency (*p* = 0.009), auditory comprehension (*p* = 0.022), repetition (*p* = 0.028), grammar (*p* = 0.005) and speech act (*p* = 0.004) scores increased statistically significantly in the moderate stage study group, while repetition (*p* = 0.028) scores decreased statistically significantly in the control group. The grammar (*p* = 0.003) score of the severe stage study group participants increased statistically significantly (Table 3).

### Participants’ application evaluation questionnaire and application factor outcomes

**FIGURE 7:**
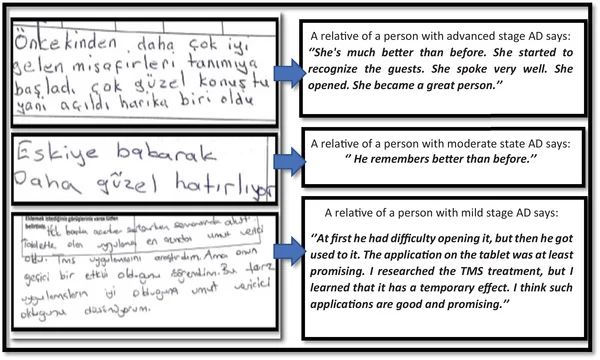
Additional statements from some participants’ caregivers. [Colour figure can be viewed at wileyonlinelibrary.com]

The relatives of all participants strongly agreed that the main sections and subsections in the application were easily accessible, the texts were in a readable size and colour, the visuals were simple and understandable, the colours used were easy on the eyes, the instructions were clear and understandable and the feedback given in the application was liked. Table 4 shows the distribution of responses to the questionnaire given to caregivers. The mean total score of the Cognitive Communication Disorder Practice Evaluation questionnaire for all participants was calculated to be 63.50 ± 2.30. There were no statistically significant differences in total and subdimension questionnaire values between the AD stages (*p* > 0.05) (Table 5). Additional statements from some patient relatives are shown in Figure 7.

**TABLE 4:** Participants’ application evaluation questionnaire outcomes.

Application evaluation questionnaire	5: Strongly agree, *n* (%)	4: Agree, *n* (%)	3: Undecided *n* (%)	2: Disagree, *n* (%)	1: Strongly disagree, *n* (%)
**I can easily log in to the application**.	14 (87.5)	2 (12.5)	–	–	–
**The application is easy to use**	15 (93.8)	1 (6.3)	–	–	–
**Even someone who has never used a tablet before can use the app**	15 (93.8)	1 (6.3)	–	–	–
**I can easily access the main sections and subsections within the application**	16 (100.0)	–	–	–	
**The texts in the application are in a readable size and colour**	16 (100.0)	–	–	–	–
**The visuals in the application are simple and clear**	16 (100.0)	–	–	–	–
**The sounds in the application are understandable and audible**	14 (87.5)	–	–	2 (12.5)	–
**The colours used in the application are easy on the eyes**	16 (100.0)	–	–	–	–
**The instructions in the application are clear and understandable**	16 (100.0)	–	–	–	–
**I liked the feedback given in practice**	16 (100.0)	–	–	–	–
**I was happy to think that something was being done for the patient**	15 (93.8)	1 (6.3)	–	–	–
**There were positive changes in the patient compared to the past**	9 (56.3)	2 (12.5)	4 (25.0)	1 (6.3)	–
**I recommend that other patients use it**	16 (100.0)	–	–	–	–

**TABLE 5:** Participants’ application factors outcomes.

Application factors (total participants; mean ± SD)	Groups			Total participants	
	Mild stage, ean ± SD	Moderate stage, mean ± SD	Severe stage, mean ± SD	*F*	*p*
**Application's ease of use (19.75 ± 0.57)**	19.83 ± 0.40	19.50 ± 0.83	20.00 ± 0	1.000	0.394
**Design (10.00 ± 0)**	10.00 ± 0	10.00 ± 0	10.00 ± 0	–	–
**Understandability (19.62 ± 1.02)**	20.00 ± 0	20.00 ± 1.54	20.00 ± 0	2.031	0.171
**Satisfaction factors (14.12 ± 1.14)**	14.50 ± 1.22	14.00 ± 0.89	13.75 ± 1.50	0.534	0.598
**Total score (63.50 ± 2.30)**	64.33 ± 1.63	62.50 ± 3.14	63.75 ± 1.50	0.973	0.404

## DISCUSSION

In line with the first aim of our study, for the first time in Turkish, mobile‐compatible software has been developed for both cognitive and communication disorders and is specific to individuals with AD. The application we developed is also the first in the international literature, as it is suitable for all stages of AD and includes a holistic cognitive–communication intervention program. With the application we developed, both communication support and therapy services are provided together. The application we developed includes the use of alternative and supportive communication, especially for individuals with severe stage AD. The effects of the application we developed in line with the other aim of our study on the cognitive–communication skills of individuals with AD were shown in a randomised controlled trial. As another aim of the study, the changes between participants in the mild, moderate, and severe stages of the study and control groups were compared, and the areas showing the most change in each stage were identified.

### Sociodemographic characteristics

It has been reported that individuals with AD may be confused with normal cognitive decline in the ageing process and even with other diseases associated with ageing, which may cause delays in patient diagnosis, and they can usually be diagnosed in the moderate stage (Sánchez et al., 2016). In our study, as in other studies, individuals who met the inclusion criteria were mostly in the moderate stage, with the fewest in the severe stage. Individuals in the mild stage may not want to come to the hospital because of their rejection of the disease, and individuals in the severe stage may have difficulties being brought to the hospital because of their increasing level of dependency (Gürvit, 2004). Therefore, in our study, it is thought that mostly individuals with moderate stage AD visited the Dementia Outpatient Clinic in the last 3 months. In our study, 67.31% of the individuals who met the inclusion and exclusion criteria were female. As a result of randomization, 59.4% of the participants were women, which is consistent with the literature. In the literature, it is stated that the incidence of AD increases with increasing age (Lin & Doraiswamy, 2015) and that the percentage of women is higher because women have a higher survival rate (Lin & Doraiswamy, 2015; Öztürk & Karan, 2009).

Studies have shown that educational status affects cognitive reserve and age affects cognitive decline (Alzheimer's Association, 2018; Cholerton et al., 2013). Aguirre et al. (2013) reported in their study that increasing age may affect the increase in MMSE scores of cognitive stimulation therapy, on which our application is based (Aguirre et al., 2013). Because of this, it is very important that the groups being compared have the same level of education and age. In our study, no statistical difference was found between the stages of AD and between the study and control groups in terms of the age, gender and education levels of the participants. As a result, cognitive communication skills are thought to have been unaffected by the comparison, and group homogeneity was ensured. Thus, it was ensured to focus only on the effect of tablet use on the change in the cognitive–communication skills of the participants.

### Cognitive status

Acetylcholinesterase inhibitors have been shown in studies to slow the rate of cognitive decline in AD. According to Mohs et al. (2001), 1 year of donepezil treatment was associated with a 38% lower risk of functional decline when compared with placebo (Mohs et al., 2001). Because there is a general agreement about the benefit of acetylcholinesterase inhibitors in AD, it is stated that analysing the effect of cognitive stimulation alone is incorrect (Mohs et al., 2001). As a result, the control group in our study consisted of people with AD who were receiving drug treatment, and there was no significant change in the MMSE total score after 7 weeks in the control group individuals who were only given acetylcholinesterase inhibitor medication. This is an expected result unless there is a rapid cognitive decline. Because the expected average decrease in the MMSE score of individuals with AD varies between 2 and 4 points per year, a loss of 4 points or more in MMSE scores in 6 months is interpreted as rapid cognitive loss (O'Hara et al., 2022; Holmes & Lovestone, 2003).

In our study, we found that the developed application increased general cognitive functions by affecting the development of orientation skills for mild and moderate individuals, language skills for moderate and advanced individuals, and attention skills only for mild individuals. Cognitive stimulation therapy has been used a lot in the past to help people with mild AD. In these studies, an increase was observed in the general cognitive functions of individuals with AD (Lök et al., 2020; Spector et al., 2010; Woods et al., ; McDermott et al., 2019), but the increase in MMSE scores was not statistically significant in some of the studies (Gibbor et al., 2021; Hall et al., 2013). They attributed this to the fact that the MMSE has a relatively small range and is less sensitive to smaller changes in cognition (Gibbor et al., 2021). In the study group including all stages of our study, MMSE total scores increased from 15.38 ± 5.80 to 19.56 ± 5.76. In addition, when analysed on a stage basis, there was a significant increase in MMSE total scores in all stages. Although it is stated to be less sensitive, it is an important result that the MMSE total score of the participants in all stages who used the application in our study increased. because it demonstrated that using the application may reduce cognitive decline. It is thought that this result may be due to the fact that the application provides multidirectional sensory input to individuals with AD, including auditory, visual and tactile input, since it includes a multidirectional section.

Bayles (2020) states that sufficient stimulation is required to trigger neuroplasticity in individuals with AD and that attention is necessary for learning during this stimulation (Amir et al., 2006). While there was a decrease in the attention and calculation section in the mild stage control group, there was a significant increase in the study group. This result showed that the use of the application may be important for cognitive functions that may have a high rate of deterioration in patients with a mild stage. Because attention is required for all cognitive functions, improvements in attention skills may help to develop other cognitive functions later in the process or slow the rate of deterioration. For this reason, it is thought that the significant change in attention skills in mild stage individuals with AD from the use of the application may be important for the development of other cognitive functions.

After the use of the application, there was a statistically significant difference in the orientation section between the study group, including mild, moderate, and all stages, and the control group. This situation suggests that the cognitive area that the application has the most effect on is orientation. The significant increase in orientation scores after the use of the application in mild and moderate stage individuals also supported this idea. This result is thought to be due to the development of person, place, and time orientation in individuals with AD by using the orientation section three times a day. In addition, in our study, a significant increase was found in the language score of the MMSE in the final evaluation compared with the initial evaluation in the study group, including moderate, severe stage and all individuals. A statistically significant difference was found between the study and control groups in the language section for all individuals after the use of the application. These results suggest that our intervention had the most effect on the language section of the MMSE after orientation.

### Communication status

It was observed that the developed application was effective in improving speech fluency and auditory comprehension for mild and moderate participants, grammar for all participants and repetition and speech act skills only for moderate participants. Chapman et al. (2004) reported that there was a positive change in the language skills of patients after cognitive stimulation therapy and that these changes occurred over a long period of time (Chapman et al., 2004). In our study, an increase was observed in the language scores of the participants after 7 weeks, according to both MMSE and LATA results. In the application we developed, having both visual, auditory and written stimuli may have increased the duration of information utilization and contributed to an increase in both MMSE and LATA scores. Similarly, the positive change in all LATA areas except writing may have resulted from the multifaceted subsections of the application. In addition, improvements in spoken language, word finding, naming and comprehension are all likely to help communication, speech and expression. The mean LATA scores of all participants in the study group who used the application increased from 198.63 to 223.18. This shows that the intervention had an effect on the participants’ language and communication skills. It is thought that not only the language section but also all sections such as simultaneous auditory memory, attention and executive function may have an effect on this increase. It is thought that language is also a cognitive function, and cognitive functions are interrelated and interconnected; therefore, the increase in cognitive scores may affect the increase in language scores.

Grammar was the only part of LATA that showed a significant increase in all phases and among all participants. For this reason, it was thought that the most beneficial part of the application was grammar. In addition, it was observed that the grammar section showed a significant difference between the study, which included participants at all stages and the control group. This is another finding that supports our decision not to consider grammar as the language area that the intervention had the most effect on. It is thought that, especially, the language section in the application and problem solving in the executive functions section have an effect on this result. Auditory attention and focusing of attention are also required for the grammar section of the LATA. Because a sentence is read to the individual in the grammar section, and the individual needs to complete this sentence. For this reason, it is thought that the attention section in the application may be another factor affecting this result. In addition, it has been reported that grammar can be preserved in some individuals until the last stages of the disease and deteriorate later (Bayles et al., 2020). Therefore, it is thought that the rate of deterioration of grammar may be less and the stimulability of this part may be higher with therapy. After grammar, a significant increase was found in auditory comprehension and speech fluency scores in both mild and moderate individuals. Therefore, it is thought that auditory comprehension and speech fluency are the other areas where the intervention may have the most effect. This idea was supported by the significant increase in speech fluency and auditory comprehension in all stage participants in the study group.

In the mild stage control group, a decrease was observed in the repetition, speech act and LATA total scores, while in the moderate stage, a decrease was observed in the repetition scores. The decrease in repetition scores in both mild and moderate participants suggests that this is the language component that can be affected most rapidly in patients. Auditory attention and auditory memory are very important for repetition. From the mild stage onward, even if patients understand most of what they hear, they rapidly forget the information, and this is the main problem for individuals in the mild stage (Bayles et al., 2020). Therefore, it is thought that repetition skills may be the area showing the fastest decline. There was a significant increase in repetition scores in the study group at the moderate stage and in all participants. This led us to think that the language area with rapid deterioration could be preserved and even improved with the use of the intervention.

### Application

In their study, Zmily et al. (2014) reported that individuals with early‐stage AD were able to use mobile devices despite having no previous experience (Zmily et al., 2014). In line with this study, in our study, it was observed that both individuals with AD and their caregivers could easily use the application, especially in the mild stage, even if they did not have application experience. Alm et al. (2004) used digital communication support for people with dementia and found that patients adjusted quickly. They also found that patients could talk about wider topics with digital communication support and that the patient and their conversation partner spoke more symmetrically with the multimedia support (Alm et al., 2004). Similar to this study, in our study, some of the relatives of the patients stated that they talked more than before and participated in the conversation. Some caregivers also said that the person with AD, even at the most severe stage, recognised people better than before, spoke better and could start and keep conversations better than before. These opinions of the relatives of the patients are important in terms of the effects of the practice. This result may have been influenced, especially by the communication board and functional expressions sections in the application. Another reason may be that the improvement in cognitive functions supports the initiation and maintenance of communication. In addition, it is thought that the expansion of past memories with the reminiscence section and the expansion of conversation topics with the recollection of individuals may also have affected the situation.

According to Samuelsson and Ekström (2019), caregivers and individuals with dementia found the use of CIRCA to be enjoyable and educational. Individuals with dementia were also interested in the tablet and wanted to participate in its use (Samuelsson & Ekström, 2019). In our study, it was discovered that people with mild to moderate AD were more willing to use the tablet, but all caregivers recommended the application for use by other patients. According to Samuelsson and Ekström (2019), digital communication support improves conversations involving people with dementia by making it easier for them to take speaking initiatives and contribute to conversation topics (Samuelsson & Ekström, 2019). Our study's findings support this viewpoint. Allowing people to talk again may improve relationships with family and friends, as well as caregivers’ quality of life (Woods et al., 2006). The quality of life of caregivers was not assessed in our study. Future research will look into the quality of life of caregivers of people with Alzheimer's who use the app.

Abu Hasim et al. (2018) created an individualised application based on reminiscence and cognitive stimulation, similar to what we did in our study. As a result of this research, they concluded that their application was appropriate for the non‐pharmacological treatment of people with AD. However, the application's effectiveness was only tested in one case (Abu Hashim‐de Vries et al., 2018). There are reminiscence and orientation sections in our application that are prepared using personal information and photographs. As is common in many therapy effectiveness studies with people with AD, our study was conducted as a randomised controlled trial with a number of people determined by power analysis. This is thought to increase the level of evidence of our application's effect. In addition, in the survey questions, the caregivers of individuals with AD at all stages, including the severe stage, stated that they would recommend the application to other patients. All these results suggested that the application was positive and that the usability of the application was high.

### Limitations

We acknowledge that our study has some limitations. One of them is that in the preparation of the content of the application, the suggestions of the families of individuals with dementia were not taken into consideration, except for one researcher who has a relative with AD. The other limitation in our study was that the participants were chosen at random to use the application. Not every person with AD and/or caregiver is eligible to use the app, and even if they are, they may not use it on a regular basis. The duration of use by participants at home is not visible in our application, and the actual duration of use is not clearly known. This situation constitutes the application's limitation. It is planned to upload the application to the Google Play Store in the future to track the duration of use and the progress of the patients. We believe that another limitation of our study is that the photographs used in the naming section were uploaded ready‐made in the application and that the participants did not have familiar photographs of their own lives. Because, although there was a special section to improve naming skills, there was not as significant an increase as we wanted. For this reason, it is planned to organise this section in the application so that each family can change the photo and upload it themselves. Despite this, the average of all caregivers’ responses to the application evaluation questionnaire was 63.50 out of 65. This subjective result suggests that the application is successful because it is supported by positive changes in objective outcomes such as LATA and MMSE in people with AD. There are very few application‐based studies in the literature, and these studies had a small sample size. There is a lack of long‐term patient follow‐up in other application‐based studies (Ye et al., 2021). One of the general limitations of cognitive stimulation therapy studies, as in ours, is the lack of long‐term follow‐up (Spector et al., 2003). Therefore, in future studies, individuals with AD can be assessed 3, 6 and 1 years after using the app, and the application's long‐term effects can be calculated. In addition, we did not collect information about caregivers in this study. In future studies, demographic information about caregivers can be obtained, and their quality of life and care burden can be examined with the application.

## CONCLUSIONS

In this study, for the first time, a mobile‐compatible application was developed for cognitive–communication disorder in individuals with AD that can be used by individuals at all stages of the disease. With the application we developed, individual therapy services were provided from home for individuals with AD. This may be a facilitator, especially for individuals with AD who have ambulation problems such as reaching the hospital and getting up and walking. In the MMSE sections of the application, it was observed that the application increased general cognitive functions by affecting the development of orientation skills for individuals with mild and moderate AD, language skills for individuals with moderate and advanced AD, and attention skills only for individuals with mild AD. It was observed that the developed application was effective in improving speech fluency and auditory comprehension for mild and moderate participants, grammar for all participants and repetition and speech act skills only for moderate participants. It was determined that the areas where the application provided the most benefit were grammar in LATA and orientation in MMSE. In this study, it was seen that it may be important to provide therapy in the early stages of AD, which is a neurodegenerative disease. Positive changes in cognitive–communication skills were found in all participants at all stages, including the severe stage. So, using the app can help people with all stages of AD have a better prognosis and keep their cognitive and communication skills.

## CONFLICT OF INTEREST STATEMENT

The authors declare no conflicts of interest.

## Data Availability

All data generated or analysed during this study are included in this article. Further enquiries can be directed to the corresponding author.
